# Properties of Heat-Treated Wood Fiber–Polylactic Acid Composite Filaments and 3D-Printed Parts Using Fused Filament Fabrication

**DOI:** 10.3390/polym16020302

**Published:** 2024-01-22

**Authors:** Yu-Chen Chien, Teng-Chun Yang

**Affiliations:** Department of Forestry, National Chung Hsing University, Taichung 402, Taiwan; g111033208@smail.nchu.edu.tw

**Keywords:** wood fiber, heat treatment, 3D printing, fused filament fabrication, wood–PLA composite (WPC), physic and mechanical properties

## Abstract

Wood fibers (WFs) were treated at a fixed heat temperature (180 °C) for 2−6 h and added to a polylactic acid (PLA) matrix to produce wood−PLA composite (WPC) filaments. Additionally, the effects of the heat-treated WFs on the physicomechanical properties and impact strength of the WPC filaments and 3D-printed WPC parts using fused filament fabrication (FFF) were examined. The results revealed that heat-treated WFs caused an increase in crystallinity and a significant reduction in the number of pores on the failure cross section of the WPC filament, resulting in a higher tensile modulus and lower elongation at break. Additionally, the printed WPC parts with heat-treated WFs had higher tensile strength and lower water absorption compared to untreated WPC parts. However, most of the mechanical properties and impact strength of 3D-printed WPC parts were not significantly influenced by adding heat-treated WFs. As described above, at the fixed fiber addition amount, adding heat-treated WFs improved the dimensional stability of the WPC parts and it enabled a high retention ratio of mechanical properties and impact strength of the WPC parts.

## 1. Introduction

3D printing technology has evolved beyond the layer-by-layer fabrication of three-dimensional structures based on computer-aided design (CAD) drawings [1]. This technology has emerged as a versatile option to overcome product processing restrictions and improve manufacturing efficiency [2]. Fused filament fabrication (FFF), which is also known as fused deposition modeling (FDM), is a popular desktop 3D printer. The main advantages of an FFF printer are the simple structure of the device, low cost, low failure rate, and ease of transportation [3,4]. Additionally, the FFF printer is suitable for use in an office due to its dust-free and low-noise operation. For the FFF printer, the polymeric filament is fed through the heated nozzle as a raw printing material to build up the desired structure. Polylactic acid (PLA) is one of the most widely used materials for FFF printing due to its biodegradability, low melting point, and low coefficient of thermal expansion. However, PLA is difficult to process due to its brittleness and hardness, and it is more expensive than petroleum-based plastics. Several previous studies reported that natural fiber-added polymeric composites have high processability, cost-effectiveness, renewability, and biodegradability [5,6].

Among various natural fibers, wood fibers are widely added as fillers to polymeric matrices to produce wood–plastic composites with low density and highly specific mechanical properties [7]. According to a review published by Das et al. [8], polymeric filaments with wood fibers exhibit low deformation and high rigidity, but are accompanied by high porosity and low mechanical properties. Kariz et al. [9] investigated the influence of wood fiber (WF) content (0–50 wt%) on the properties of wood–PLA composite (WPC) filaments. The results showed that the WPC filament with 10 wt% WFs had the highest tensile strength, whereas a decrease in the density and an increase in the roughness on the surface of the filament were noted as the WF content increased. Le Duigou et al. [10] found that printing orientation and width affect the water absorption and tensile properties of FFF-printed WPC parts, and they printed a WPC part with a bilayer microstructure to produce hygromorphic biocomposites. Le Guen et al. [11] explored the rheological behavior of PLA filaments with 10 wt% biofillers (rice husks and WFs) and the mechanical properties of printed parts. They demonstrated that while the addition of WF increased the complex viscosity, there were no significant differences in the mechanical properties among all the filaments. Fico et al. [12] characterized the life cycle assessment (LCA) and physical, thermal, and mechanical properties of WPC filaments with different amounts of olive wood scraps (10–20 wt%) and FFF-printed parts. They indicated that the addition of WFs increased the crystallinity of the PLA matrix, while it caused a decrease in the flexural properties and the hardness of 3D-printed WPC bars. Additionally, their LCA results indicated that the environmental benefits from the effective utilization of WFs for a 3D printing filament could be an eco-friendly solution. According to the forest resources survey reported by the Forestry and Nature Conservation Agency in Taiwan [13], Japanese cedar (*Cryptomeria japonica* D. Don) is the main species in Taiwan’s coniferous artificial forests. In 2020, its forest land area was about 30,555 ha, accounting for nearly 33%. Therefore, Japanese cedar was used as a filler to fabricate WPC filaments in the present study.

However, it is well known that the main drawbacks of WPCs are attributable to WFs being hydrophilic and polar in nature; these drawbacks are dimensional instability, incompatibility between the fibers and the matrix, nonuniform dispersion of fibers, and low thermal stability [14]. Sodium hydroxide (NaOH) treatment, which is one of chemical approaches, is being widely used to modify the lignocellulosic materials [15]. Through this treatment, mechanical and thermal properties of the composite with NaOH-treated fibers are significantly improved, and good adhesion between the fibers and the matrix is observed [16,17,18]. However, this chemical modification is not eco-effective due to being a chemically-based method, time consuming, and having chemical waste produced after treatment. Therefore, heat treatment, which is a low-cost, physical, and eco-friendly modification, has been attractive in various fields. Many studies have indicated that WFs treated by heat treatment could improve the water resistance and thermal stability of WPCs and enhance interfacial compatibility between WFs and the polymeric matrix [14,19,20]. To date, a WPC with heat-treated WFs for 3D printing filaments has not yet been reported in the literature. In general, the temperature range of heat treatment for lignocellulosic materials is from 150 to 230 °C [14,21,22]. Previous studies reported that water absorption of bamboo or wood treated at 170–180 °C significantly decreased and there is no significant difference for their mechanical properties compared to untreated ones [14,21,22]. Accordingly, the surface morphology, crystallinity by DSC analysis, and tensile properties of the WPC filaments with WFs treated at the fixed temperature of 180 °C for different levels of heat treatment time (2–6 h) under air were explored in the present study. Furthermore, the surface color, dimensional ability (water absorption and thickness swelling), mechanical properties (tensile properties and flexural properties), and impact strength of the heat-treated WPC parts using FFF were also investigated.

## 2. Materials and Methods

### 2.1. Materials and Heat Treatment Process

Polylactic acid (PLA) as a polymeric matrix was purchased from Color Matrix Co., Ltd., Taichung, Taiwan, and its melting temperature was 176 °C. Japanese cedar (*Cryptomeria japonica* D. Don) sapwood was obtained from the experimental forest of National Taiwan University, Nan-Tou County, Taiwan. Sapwood was milled and sieved with an Ultra Centrifugal Mill ZM-1 (Retsch GmbH, Haan, Germany) to prepare wood fibers (WFs) with a size below 100 mesh. For heat treatment, the WFs were heated at a fixed temperature of 180 °C for 2–6 h under air in a conventional oven (JB-27, ProKao Instrument Co., Taichung, Taiwan).

### 2.2. WPC Filaments and 3D-Printed WPC Parts

**Figure 1:**
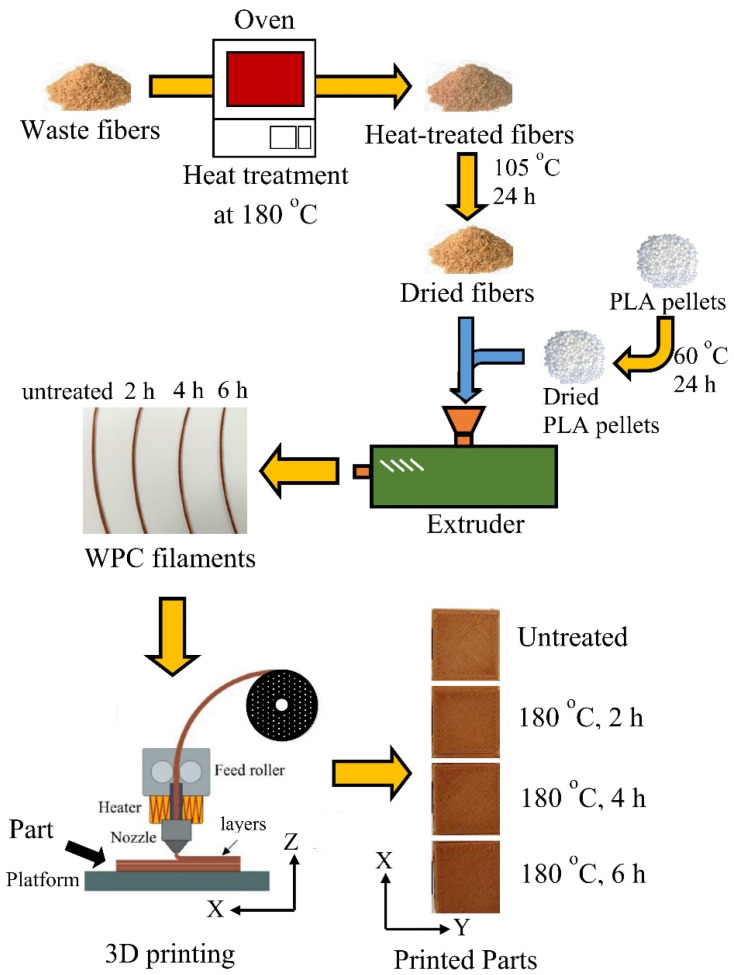
Schematic diagram of the manufacturing of WPC filaments and 3D printing of WPC parts.

The WFs and PLA pellets were dried at 105 °C and 60 °C for 24 h prior to mixing. The weight ratio of WFs to PLA was 20/80. As shown in Figure 1, the various ingredients were mixed to produce the WPC mixtures using a single-screw extruder (EX6 Filament Extruder, Filabot Co., Ltd., Barre, VT, USA) at a screw speed of 16 rpm. The temperatures from the feed zone to the melting/pumping zone were 70, 210, 180, and 176 °C. To increase the homogeneity of the filament, the WPC mixtures were extruded twice to obtain WPC filaments (WPC_F_s) with a diameter of 1.65 ± 0.1 mm. All WPC parts (WPC_P_s) with a layer thickness of 0.3 mm were fabricated using an FFF printer (Creator Pro, Flashforge 3D Technology Co., Ltd., Jinhua, China) with a 0.6 mm nozzle size. According to the sample shapes for various tests, all the samples were printed to orient parallelly along the printing axis (X-axis) with a 100% filling pattern, and a printed contour was added around the test sample. The temperatures of the nozzle and heating plate were 210 °C and 60 °C, respectively. Additionally, the printing speed was set to 30 mm/s. All the samples were conditioned at 20 °C and 65% relative humidity (RH) for 1 week.

### 2.3. Properties of WPC Filaments

DSC analysis of the filament with 3.5–5 mg was recorded using a PerkinElmer DSC-6 (Beaconsfield, UK) at a flow rate of 20 mL/min under nitrogen. The filament was heated from 20 °C to 210 °C at a heating rate of 10 °C/min. The crystallinity was calculated according to the following equation: X_c_ (%) = 100 × (ΔH_m_ − ΔH_cc_)/(ΔH^o^_m_ × w_c_), where ΔH_m_ and ΔH_cc_ refer to the enthalpies of melting and cold crystallization, ΔH^o^_m_ refers to the enthalpy of melting of 100% crystallized PLA (93 J/g), and w_c_ refers to the weight fraction of the PLA matrix in the WPC. Additionally, the surface morphology and failure cross-sectional surface of WPC filaments with different heat-treated WFs were obtained from SEM micrographs using a Hitachi TM–1000 (Tokyo, Japan) with an acceleration voltage of 15 kV. For tensile properties, the tensile strength (TS_F_), tensile modulus (TM_F_), and elongation at break (EB_F_) of the WPC filaments were assessed with a span of 30 mm at a loading speed of 5 mm/min.

### 2.4. Properties of 3D-Printed WPC Parts

#### 2.4.1. Surface Color

The CIE L*a*b* color system on the surface color of the printed WPC part was measured by a UV–Vis-NIR spectrophotometer (LAMBDA 1050+, PerkinElmer Co., Ltd., Waltham, MA, USA) in the spectral range of 380–780 nm. The color difference (ΔE*) was determined as ΔE* = [(ΔL*)^2^ + (Δa*)^2^ + (Δb*)^2^]^1/2^, where L* is the value on the white/black axis, a* is the value on the red/green axis, and b* is the value on the yellow/blue axis.

#### 2.4.2. Physical and Mechanical Properties

Physical properties, including density, moisture content (MC), water absorption (WA), and thickness swelling (TKS), of the printed WPC part were determined in this study. According to CNS 13333-1 [23], the density of the printed part (sample size: 10 mm (X) × 10 mm (Y) × 5 mm (Z)) was estimated using the Archimedes method with a semimicro analytical balance (GH-200, A&D Co., Ltd., Tokyo, Japan). According to ASTM D4442-20 [24], the MC value of the sample (sample size: 80 mm (X) × 12 mm (Y) × 3 mm (Z)) was assessed. According to ASTM D1037-12 [25], all the printed samples were previously oven-dried at 60 °C for 72 h. Afterward, the samples were fully immersed in water at 23 °C for 24 h, and the weight and thickness were recorded to calculate the WA and TKS values of the printed WPC parts. Using ASTM D638-14 [26] for the tensile test, the tensile strength (TS), tensile modulus (TM), and elongation at break (EB) of the printed WPC part with type IV were assessed at a loading speed of 5 mm/min and a span of 65 mm. For the flexural test, according to ASTM D790-17 [27], the modulus of rupture (MOR) and modulus of elasticity (MOE) were obtained using a three-point bending test at a span of 48 mm and a crosshead speed of 1.28 mm/min (sample size: 80 mm (X) × 12 mm (Y) × 3 mm (Z)).

#### 2.4.3. Impact Strength

**Figure 2:**
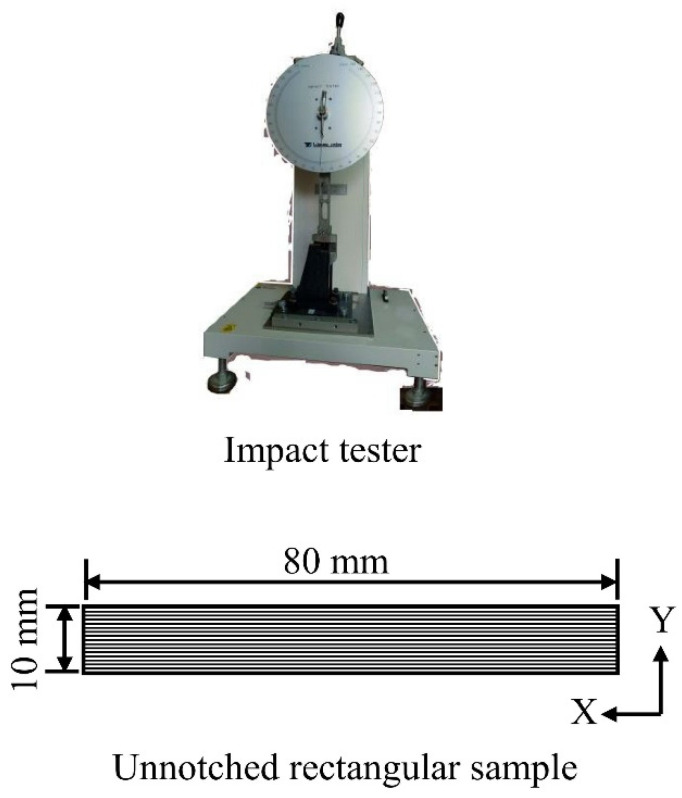
Appearances of an impact tester and an unnotched impact sample.

The Charpy impact strength (IS) was evaluated by testing 5 unnotched rectangular samples (sample size: 80 mm (X) × 10 mm (Y) × 4 mm (Z)) per printed WPC part using a YASUDA Impact Tester (Nishinomiya, Japan) according to CNS 5846-1 [28] (Figure 2).

### 2.5. Analysis of Variance

The significance of the differences among all the samples was calculated using Scheffe’s test (*p* < 0.05). Additionally, significant difference was investigated for each property of the heat-treated WPC sample and untreated WPC sample using Student’s *t*-test (*p* < 0.05).

## 3. Results and Discussion

### 3.1. Properties of WPC Filaments with Heat-Treated WFs

The tensile properties of WPC filaments (WPC_F_) with different heat-treated WFs are presented in Table 1, including the tensile strength of a filament (TS_F_), tensile modulus of a filament (TM_F_), and elongation at break of a filament (EB_F_). The TS_F_ value of the WPC filament with untreated WFs (WPC_FNT_) is 44.3 MPa, while the TS_F_ values of the WPC filaments with heat-treated WFs are in the range of 41.4 to 47.0 MPa. In the statistical analysis, there were no significant differences among all the TS_F_ values of the WPC_F_s. This result indicated that the tensile strength of the WPC_F_ was not affected by adding WFs treated at various treatment times. According to previous studies [14,29,30], the main reason for this phenomenon is the mutual offset between the reduction in fiber strength due to heat treatment and the improvement in compatibility between the fibers and the matrix. Additionally, the TM_F_ value of the WPC_FT4_ significantly increased from 3.2 (WPC_FNT_) to 3.7 GPa. This is related to the increased compatibility of the fiber/matrix interface for the WPC_F_ with heat-treated WFs compared to that with untreated WFs.

**Figure 3:**
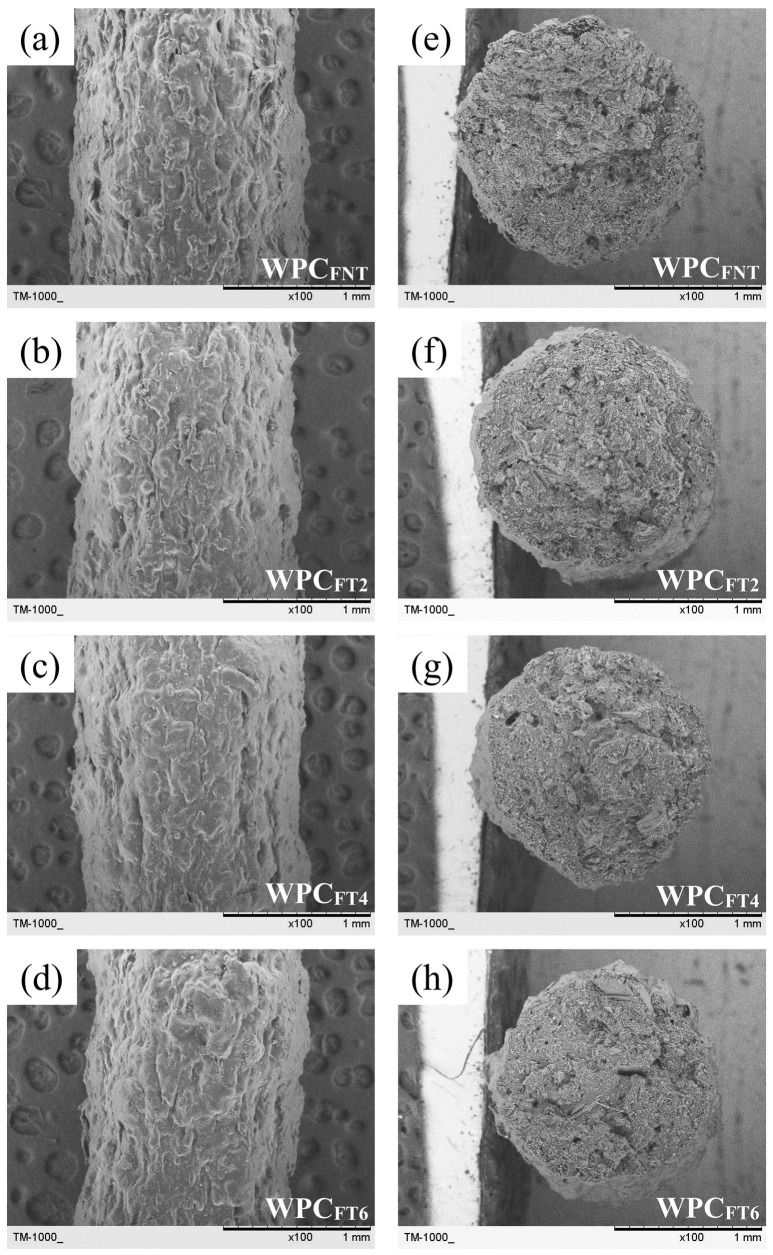
(**a**–**d**) Surface morphology and (**e**–**g**) failure cross-sectional surfaces of WPC filaments with different heat-treated WFs. (**a**,**e**): WPC_FNT_; (**b**,**f**): WPC_FT2_; (**c**,**g**): WPC_FT4_; (**d**,**h**): WPC_FT6_.

**Figure 4:**
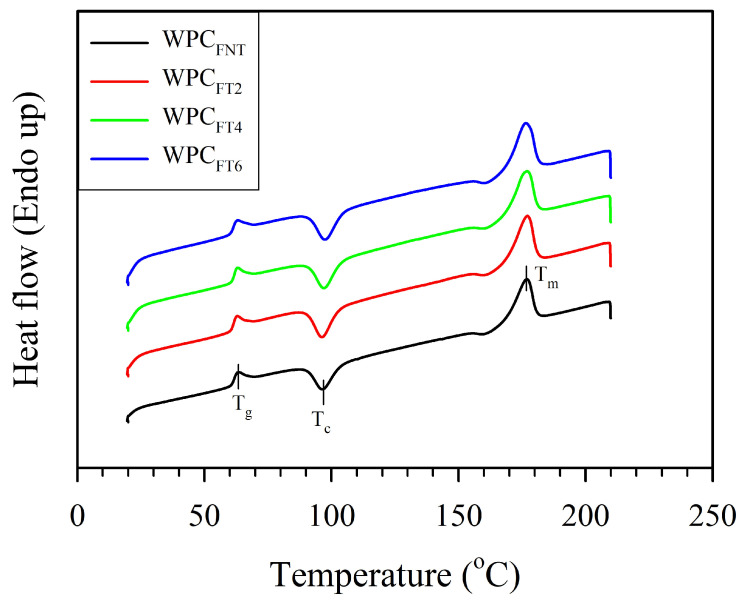
Heat flow of WPC filaments with different heat-treated WFs.

Figure 3 displays the surface morphology and failure cross-sectional surfaces of WPC filaments with WFs treated at various treatment times. After the addition of untreated WFs, the surface morphology of the WPC_Fs_ became uneven (Figure 3a), and several pores were observed on the cross section (Figure 3e). Previous studies [12,31] reported that the PLA matrix with a nonpolar surface and WFs with a polar surface led to poor interfacial adhesion, further resulting in fiber agglomerations, nonuniform dispersion of fibers in the PLA matrix, and several pores produced by fibers pulled out from the PLA matrix. Compared to the WPC_FNT_, the heat-treated WPC_F_ exhibited a smooth surface morphology and a significant reduction in the number of pores on the failure cross section. The results confirmed that the improvement in fiber–matrix adhesion caused a corresponding increase in the TM_F_ value, especially for WPC_FT4_. To investigate the effect of heat-treated WFs on the phase transitions in the PLA matrix, a thermal analysis of the WPC filaments was performed using DSC measurements. Figure 4 shows the curves for the heat flow of WPC filaments with different heat-treated WFs. No obvious changes were observed for any of the samples during the melting process. As shown in Table 2, the glass transition temperature (T_g_), cold crystallization temperature (T_cc_), and melting temperature (T_m_) are listed, and the crystallinity degree (X_c_) is calculated from the DSC curves of the WPC filaments. Regardless of whether the fibers were untreated or heat-treated, the T_g_ and T_m_ values were in the ranges of 61.5–61.9 °C and 176.4–176.9 °C, respectively. Additionally, the T_cc_ value slightly increased from 96.7 (WPC_FNT_) to 97.6 °C (WPC_FT6_) when the treatment time reached 6 h. This result indicated that the nucleating ability of heat-treated WFs increased with an increase in treatment time. Furthermore, the X_c_ value for WPC_FNT_ was 23.4%, while the addition of heat-treated WFs to the PLA matrix increased the X_c_ value in the range of 34.0–43.9%, with the use of WFs treated at greater treatment times resulting in higher values. Odalanowska and Borysiak [32] reported that a significant increase in nucleation activity of the WF surface was estimated in WPCs with WF after heat treatment in the temperature range of 160–180 °C. This is due to thermal degradation of the most unstable chemical composition in this temperature range, such as hemicelluloses. This change allows cellulose fibers to freely arrange their structures with greater orderliness, being further conducive to forming transcrystalline structures and crystal growth [32]. Therefore, the heat-treated WPC_F_ had a higher TM_F_ value and lower EB_F_ value due to the higher degree of crystallinity in the PLA matrix (Table 1 and Table 2). According to previous studies [33,34], an increase in the crystallinity of the polymeric matrix increases the mechanical strength and modulus of composites but reduces the elongation at break. In the present study, the EB_F_ value of the WPC_F_ significantly decreased from 3.0 to 1.9% when the treatment time reached 6 h (WPC_FT6_). Except for the higher crystallinity for the WPC_F_ with heat-treated WFs (Table 2), this may be mainly attributed to the higher weight of the WFs for the WPC_FT6_. Yang et al. [35] stated that the mass loss of the lignocellulosic material increases with increasing intensity of heat treatment, such as treatment temperature and time. Therefore, the weight of the heat-treated WFs needed to be higher to fabricate the WPC_Fs_ with the given weight ratio of the WFs, especially with a longer treatment time.

### 3.2. Properties of 3D-Printed WPC Parts

#### 3.2.1. Surface Color

**Figure 5:**
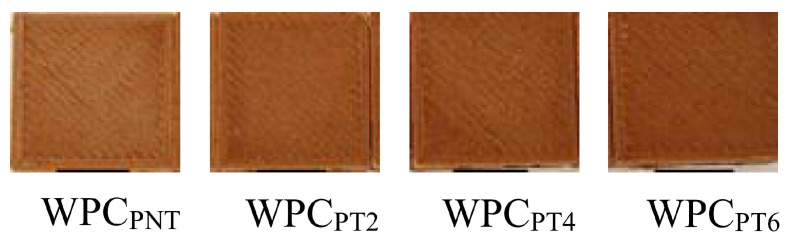
Surface appearances of 3D-printed WPC parts with different heat-treated WFs.

The surface appearances of 3D-printed WPC parts with different heat-treated WFs are illustrated in Figure 5. The color on the surface of the printed WPC part becomes darker upon adding heat-treated WFs. Table 3 shows the color parameters of 3D-printed WPC parts with different heat-treated WFs. The L* value significantly decreased from 54.3 (WPC_PNT_) to 45.6 (WPC_PT6_) with increasing treatment time. Simultaneously, the a* value increased from 10.0 (WPC_PNT_) to 11.9 (WPC_PT6_), while the b* value decreased from 25.2 (WPC_PNT_) to 24.3 (WPC_PT6_). Compared to WPC_PT4_, the color difference (ΔE*) of the printed WPC part increased to 8.9 as the treatment time increased to 6 h. The color change for heat-treated wood is attributed to the fact that hemicellulose and amorphous matter undergo depolymerization and acid hydrolysis reactions, resulting in the formation of dark-colored byproducts, such as furfural and dehydrated glucose. Additionally, lignin undergoes demethoxylation and *β*−*O*−4 bond cleavage to lead to the generation of low-molecular-weight, highly reactive, and soluble lignin, ultimately forming chromophores and auxochromes by cross-linking and condensation reactions, such as quinone compounds [36,37]. According to Bekhta and Niemz [38], darker wood browning after heat treatment is influenced primarily by changes in polysaccharides and extractives. Gaff et al. [36] and Bourgois et al. [39] reported that a decrease in hemicellulose causes a significant decrease in the L* value and a highly linear correlation between the L* value and the content of hemicellulose.

#### 3.2.2. Physical Properties

The physical properties, including density, moisture content (MC), water absorption (WA), and thickness swelling (TKS), of the 3D-printed WPC parts with different heat-treated WFs are listed in Table 4. Generally, the mechanical properties of a WPC may be directly influenced by its density and MC value. The densities and MC values were evaluated in the range of 0.99–1.06 g/cm^3^ and 1.0–1.1%, and there were no significant differences among all the printed samples. After the water absorption test for 24 h, the WA value significantly decreased from 3.9% (WPC_PNT_) to 3.2% (WPC_PT6_) as the treatment time increased. The water absorption behavior of the WPC_PNT_ is attributed to the better hydrogen bonding between water molecules and free hydroxyl groups in the cellulosic cell wall of untreated WFs [10,14,40]. Additionally, Le Duigou et al. [10] stated that the gaps at layer interfaces and pores that are produced during 3D printing promote the absorption and diffusion of water into the printed samples. The addition of the heated-treated WFs into the printed WPC part showed a lower WA value compared to the WPC_PNT_. This phenomenon is mainly ascribed to the change in the chemical composition of WFs, such as hemicellulose decomposition during heat treatment, which decreases the hygroscopicity and dimensional instability of WFs [14,20,41,42]. WPC_PT6_ showed an average TKS value (0.1%); however, it exhibited no significant difference among the samples with different heat-treated WFs. Regardless of the various heat-treated WFs, this may be due to the better wettability of the PLA matrix on the WF surfaces to sufficiently inhibit thickness swelling of the printed samples after the water absorption test [29].

#### 3.2.3. Mechanical Properties and Impact Strength

The mechanical properties and impact strength of the 3D-printed WPC parts with different heat-treated WFs are listed in Table 5. The WPC_PNT_ showed tensile strength (TS), tensile modulus (TM), and elongation at break (EB) values of 25.5 MPa, 2.7 GPa, and 1.9%, respectively. For WPC parts with heat-treated WFs, the TS value increased by 13.7% to 19.6% compared to the WPC_PNT_. No significant differences were noted for the TS values among all the WPC parts with WFs treated at different treatment times. Additionally, their TM values and EB values were in the ranges of 2.6–3.1 GPa and 1.9–2.0%. For flexural properties, the untreated and heat-treated WPC parts exhibited MOR and MOE values in the range of 48.7–52.3 MPa and 2.4–2.6 GPa, respectively. Similarly, no significant changes in the flexural properties of the printed WPC parts were noted among all the samples. These results indicated that those values of the 3D-printed WPC parts were not influenced by adding heat-treated WFs. However, the TM value of WPC_PT6_ showed a significant difference from that of WPC_PNT_ via Student’s *t*-test. As described above, the WPC parts printed with heat-treated WFs exhibited an increase in the TS value and a significant difference from the WPC_PNT_. This result implied that the heat-treated WFs improved the tensile properties of the printed WPC parts. This phenomenon is different from the trend of the tensile properties of the WPC filaments (Table 1). This finding may be related to the fact that the WPC filament (diameter: ca. 1.65 mm) feeds through the relatively narrow nozzle (diameter: 0.6 mm) to produce denser printing layers, causing significantly better compatibility between the fiber/PLA interfaces. Furthermore, the impact strength (IS) of 3D-printed WPC parts, with respect to heat-treated WFs, is shown in Table 5. The IS value of the WPC_PNT_ was 7.7 kJ/m^2^, while this value significantly decreased to 6.3 kJ/m^2^ when the treatment time reached 6 h (WPC_PT6_). According to previous studies [43,44], the impact strength of the fiber-added composite is attributed to dissipating energy during the breaking of fibers or fiber pull-out under loading. Compared to the WPC_PNT_, there was no significant difference in the IS value of each heat-treated WPC_P_. The high retention ratio of impact strength may be related to the better interfacial strength between the surface of the fiber and the matrix in the WPC_P_ with WFs treated above 6 h. Moreover, a decrease in the IS value of WPC_PT6_ is due to a significant decrease in fiber strength after a treatment time of 6 h.

## 4. Conclusions

In this study, wood–PLA composites (WPCs) with heat-treated wood fibers (WFs) were used to fabricate WPC filaments (WPC_F_s), and WPC parts (WPC_P_s) were printed using fused filament fabrication. The physical properties, mechanical properties, and impact strength of heat-treated WPC filaments and their printing parts were investigated. The results indicated that there were no significant differences in the tensile strength among all the WPC_F_s, while the tensile modulus increased and elongation at break decreased as the heat treatment time increased. A smooth surface morphology and a significant reduction in the number of pores on the failure cross section were observed for the heat-treated WPC_F_ in SEM images. From DSC analysis, the heat-treated WFs caused a higher crystallinity of PLA in the WPC_F_s, resulting in an increase in tensile modulus and a decrease in elongation at break. For the WPC_P_s, the lightness (L*) on the surface significantly decreased with increasing treatment time; however, the color difference (ΔE*) increased. After the water absorption test, the WPC_P_s with heat-treated WFs had lower water absorption than the untreated WPC_P_s, especially with the addition of WFs treated at higher treatment times. Furthermore, the tensile strength of the heat-treated WPC_P_s increased by 13.7% to 19.6% compared to that of the untreated WPC_P_s. No significant differences were noted for the tensile strength among all the WPC parts with WFs treated at different treatment times. Additionally, several tensile properties (tensile modulus and elongation at break) and flexural properties (MOR and MOE) of the 3D-printed WPC parts were not influenced by adding heat-treated WFs. For impact strength (IS), the IS value significantly decreased from 7.7 kJ/m^2^ to 6.3 kJ/m^2^ when the treatment time reached 6 h. Compared to the untreated WPC_P_, there was no significant difference in the IS value of each heat-treated WPC_P_. This result indicated that the heat-treated WPC_P_ had a high retention ratio of the IS value, even if heat-treated WFs were added.

## Figures and Tables

**Table 1:** Tensile properties of WPC filaments with different heat-treated WFs.

Code	Treatment Temperature (°C)	Treatment Time (h)	TS_F_ (MPa)	TM_F_ (GPa)	EB_F_ (%)
WPC_FNT_	-	-	44.3 ± 4.5 ^a^	3.2 ± 0.2 ^b^	3.0 ± 0.3 ^a^
WPC_FT2_	180	2	44.7 ± 4.7 ^a^_ns_	3.3 ± 0.5 ^a,b^_ns_	2.2 ± 0.4 ^a,b,^*
WPC_FT4_	180	4	47.0 ± 6.9 ^a^_ns_	3.7 ± 0.2 ^a,^**	2.2 ± 0.7 ^a,b,^*
WPC_FT6_	180	6	41.4 ± 6.7 ^a^_ns_	3.6 ± 0.1 ^a,b,^**	1.9 ± 0.6 ^b,^*

Values are the mean ± SD (*n* = 6). Different letters within a column indicate significant differences (*p* < 0.05). ns: nonsignificant; *: *p* < 0.05; **: *p* < 0.01, compared with WPC_FNT_ via Student’s *t*-test.

**Table 2:** Thermal analysis of WPC filaments with different heat-treated WFs.

Code	Treatment Temperature (°C)	Treatment Time (h)	T_g_ (°C)	T_cc_ (°C)	T_m_ (°C)	X_c_ (%)
WPC_FNT_	-	-	61.7	96.7	176.8	23.4
WPC_FT2_	180	2	61.6	96.5	176.9	34.0
WPC_FT4_	180	4	61.9	97.1	176.8	40.5
WPC_FT6_	180	6	61.5	97.6	176.4	43.9

**Table 3:** Color parameters of 3D-printed WPC parts with different heat-treated WFs.

Code	Treatment Temperature (°C)	Treatment Time (h)	L*	a*	b*	ΔE*
WPC_PNT_	-	-	54.3 ± 0.3 ^a^	10.0 ± 0.3 ^c^	25.2 ± 0.4 ^a,b^	-
WPC_PT2_	180	2	50.0 ± 0.2 ^b^	11.3 ± 0.1 ^b^	26.1 ± 0.3 ^a^	4.6 ± 0.2 ^b^
WPC_PT4_	180	4	48.8 ± 0.6 ^b^	10.9 ± 0.2 ^a,b^	25.2 ± 0.3 ^a,b^	5.6 ± 0.6 ^b^
WPC_PT6_	180	6	45.6 ± 0.6 ^c^	11.9 ± 0.5 ^a^	24.3 ± 0.6 ^b^	8.9 ± 0.7 ^a^

Values are the mean ± SD (*n* = 3). Different letters within a column indicate significant differences (*p* < 0.05).

**Table 4:** Moisture content (MC), water absorption (WA), and thickness swelling (TKS) of 3D-printed WPC parts with different heat-treated WFs.

Code	Treatment Temperature (°C)	Treatment Time (h)	Density (g/cm^3^)	MC (%)	24 h of Soaking	
					WA (%)	TKS (%)
WPC_PNT_	-	-	1.06 ± 0.02 ^a^	1.1 ± 0.0 ^a^	3.9 ± 0.4 ^a^	0.52 ± 0.18 ^a^
WPC_PT2_	180	2	0.99 ± 0.04 ^a^_ns_	1.1 ± 0.0 ^a^_ns_	4.0 ± 0.2 ^a^_ns_	0.51 ± 0.30 ^a^_ns_
WPC_PT4_	180	4	1.04 ± 0.03 ^a^_ns_	1.1 ± 0.0 ^a^_ns_	3.5 ± 0.3 ^a,b,^*	0.25 ± 0.31 ^a^_ns_
WPC_PT6_	180	6	1.02 ± 0.04 ^a^_ns_	1.0 ± 0.1 ^a,^*	3.2 ± 0.3 ^b,^***	0.10 ± 0.22 ^a^_ns_

Values are the mean ± SD (*n* = 3 for density and *n* = 5 for MC, WA, and TKS). Different letters within a column indicate significant differences (*p* < 0.05). ns: nonsignificant; *: *p* < 0.05; ***: *p* < 0.005, compared with WPC_PNT_ via Student’s *t*-test. Density is measured by CNS 13333-1 [23]. MC is measured by ASTM D4442-20 [24]. WA and TKS are measured by ASTM D1037-12 [25].

**Table 5:** Mechanical properties and impact strength of 3D-printed WPC parts with different heat-treated WFs.

Code	Treatment Temperature (°C)	Treatment Time (h)	Tensile Properties			Flexural Properties		IS (kJ/m^2^)
			TS (MPa)	TM (GPa)	EB (%)	MOR (MPa)	MOE (GPa)	
WPC_PNT_	-	-	25.5 ± 1.8 ^b^	2.7 ± 0.2 ^a^	1.9 ± 0.1 ^a^	50.3 ± 2.0 ^a^	2.6 ± 0.1 ^a^	7.7 ± 1.1 ^a^
WPC_PT2_	180	2	29.0 ± 0.8 ^a,^*	2.6 ± 0.5 ^a^_ns_	2.0 ± 0.1 ^a^_ns_	50.6 ± 2.9 ^a^_ns_	2.6 ± 0.1 ^a^_ns_	6.8 ± 0.4 ^a,b^_ns_
WPC_PT4_	180	4	30.5 ± 1.5 ^a,^*	2.7 ± 0.5 ^a^_ns_	1.9 ± 0.1 ^a^_ns_	52.3 ± 1.1 ^a^_ns_	2.6 ± 0.2 ^a^_ns_	7.1 ± 0.3 ^a,b^_ns_
WPC_PT6_	180	6	30.4 ± 0.6 ^a,^*	3.1 ± 0.1 ^a,^*	1.9 ± 0.1 ^a^_ns_	48.7 ± 3.3 ^a^_ns_	2.4 ± 0.3 ^a^_ns_	6.3 ± 0.4 ^b^_ns_

Values are the mean ± SD (*n* = 5). Different letters within a column indicate significant differences (*p* < 0.05) by the Scheffe test. ns: nonsignificant; *: *p* < 0.05, compared with WPC_PNT_ by Student’s *t* test. Tensile properties are measured by ASTM D638-14 [26]. Flexural properties are measured by ASTM D790-17 [27]. IS is measured by CNS 5846-1 [28].

## Data Availability

Data available on request from the authors.
